# Lipids, lysosomes and mitochondria: insights into Lewy body formation from rare monogenic disorders

**DOI:** 10.1007/s00401-021-02266-7

**Published:** 2021-01-30

**Authors:** Daniel Erskine, David Koss, Viktor I. Korolchuk, Tiago F. Outeiro, Johannes Attems, Ian McKeith

**Affiliations:** 1 Newcastle University Translational and Clinical Research Institute, Newcastle University, Newcastle upon Tyne, UK; 2 Wellcome Centre for Mitochondrial Research, Newcastle upon Tyne, UK; 3 Newcastle University Biosciences Institute, Newcastle University, Newcastle upon Tyne, UK; 4 Department of Experimental Neurodegeneration, Center for Biostructural Imaging of Neurodegeneration, University Medical Center Goettingen, Goettingen, Germany; 5 Max Planck Institute for Experimental Medicine, Goettingen, Germany; 6 Scientific Employee With an Honorary Contract at Deutsches Zentrum Für Neurodegenerative Erkrankungen (DZNE), Göttingen, Germany

**Keywords:** Lewy body, Alpha-synuclein, Lipid metabolism, Autophagy, Catabolism, Mitochondria

## Abstract

Accumulation of the protein α-synuclein into insoluble intracellular deposits termed Lewy bodies (LBs) is the characteristic neuropathological feature of LB diseases, such as Parkinson’s disease (PD), Parkinson’s disease dementia (PDD) and dementia with LB (DLB). α-Synuclein aggregation is thought to be a critical pathogenic event in the aetiology of LB disease, based on genetic analyses, fundamental studies using model systems, and the observation of LB pathology in post-mortem tissue. However, some monogenic disorders not traditionally characterised as synucleinopathies, such as lysosomal storage disorders, iron storage disorders and mitochondrial diseases, appear disproportionately vulnerable to the deposition of LBs, perhaps suggesting the process of LB formation may be a result of processes perturbed as a result of these conditions. The present review discusses biological pathways common to monogenic disorders associated with LB formation, identifying catabolic processes, particularly related to lipid homeostasis, autophagy and mitochondrial function, as processes that could contribute to LB formation. These findings are discussed in the context of known mediators of α-synuclein aggregation, highlighting the potential influence of impairments to these processes in the aetiology of LB formation.

## Introduction

The Lewy body (LB) diseases, including Parkinson’s disease (PD), Parkinson’s disease dementia (PDD) and dementia with LBs (DLB), are thought to lie on a clinical and pathological continuum of motor and cognitive symptoms [60]. PD presents with a rest tremor, bradykinesia and an unsteady gait, that can develop into dementia termed PDD, whilst DLB presents with cognitive impairment that can later develop into motor symptoms similar to PD [75]. All LB diseases are characterised by the accumulation of the protein α-synuclein into spherical intracellular deposits termed LBs [75, 115]. The central role of α-synuclein in LB diseases originated from the finding of mutations in the α-synuclein gene *SNCA* causing familial PD [94], and the presence of α-synuclein in LB pathology [115]. Although there is continued controversy surrounding the direct relevance of LBs to the clinical features of LB diseases (LBDs), the aggregation of α-synuclein is thought to be a critical event in the development of LBDs [88].

The native structure of α-synuclein is thought to dynamically shift between an unstructured monomer and a helically folded tetramer, with disassembly of tetramers into aggregation-prone monomers thought to be crucial for the aggregation propensity of α-synuclein [9, 25, 85]. The aggregation of α-synuclein is thought to occur in two stages, characterised initially by a nucleation phase where soluble monomers form into transient oligomers, prior to being built upon during an exponential elongation phase that produces filaments that are incorporated into fibrillary structures, such as LBs [82]. The application of exogenous fibrils to cells in culture induces the misfolding of monomeric α-synuclein, leading to accumulation of loosely organised filaments, prior to reorganisation of fibrils into spherical LB-like lesions over time [72]. However, the direct functional consequences of LB formation on cell viability remains elusive, with some studies highlighting the potential importance of an ill-defined pool of pre-fibrillar oligomers as the primary causative agents of neurodegeneration in LB disease [2], recent studies have suggested LB-like aggregates to be the primary drivers of neurodegeneration [72, 101]. Despite controversies surrounding the role of LBs in neurodegeneration in LB disease, the central role ascribed to α-synuclein aggregation in LB diseases means that understanding the genesis of α-synuclein aggregation is a pressing issue.

Although LBs are typically thought to be the hallmark pathological lesion associated with LB diseases, they are also observed in cases of several rare genetic disorders, including some forms of familial PD [8], neurodegeneration with brain iron accumulation (NBIA) [104], lysosomal storage disorders [108] and mitochondrial diseases [28]. In these conditions, the proportion of cases that manifest LBs is higher than would be expected in a comparable control population, implying a relationship between the genetic defect giving rise to the disease and LB formation. Furthermore, as LB disease and asymptomatic incidental LBs are typically only observed in elderly individuals, the young age at which LBs have been reported in some disorders implies that these are not simply incidental occurrences.

If LBs are a consequence of perturbed functioning of particular cellular pathways, then understanding the underlying cause of rare genetic disorders characterised by LBs could provide insights into LB formation. The present review will summarise the range of disorders in which LBs have been reported, and discuss how a holistic view of this disparate range of diseases may generate insights into LB formation in idiopathic LB disease. The review is not intended to be a comprehensive summary of disorders with a parkinsonian phenotype, and is focused instead on attempting to understand why LBs may form in idiopathic LBD by examining monogenic disorders with evidence of LB pathology on neuropathological examination.

## Monogenic diseases associated with α-synuclein pathology

### Familial PD

Whilst the majority of PD cases are idiopathic, a significant minority result from genetic mutations with varying patterns of clinical features and neuropathological lesion formation. An increasing number of genes have been associated with familial PD, with varying similarity to idiopathic PD, and these have been reviewed elsewhere [8]. Familial PD syndromes are highly clinically heterogenous in terms of age of onset and clinical presentation, though all typically include parkinsonian motor features, but can vary from that observed in idiopathic LBD. However, the present review will only discuss those forms of familial PD that have documented evidence of LB pathology, or a higher rate of LB pathology than would be expected in a comparable control population, and includes mutations in *SNCA* [64, 73, 92, 106, 137], *LRRK2* [98], *DNAJC13* [131], *PRKN* [103], *PINK1* [100, 116, 127], *DJ-1/PARK7* [126], TMEM230 [24] and *LRP10* [95, 129], as described in Table 1. While some, such as *PRKN*, may seem controversial as only approximately 33% of cases manifest LBs, leading to its characterisation as a primary nigropathy [27], as incidental LBs occur in only 10% of the normal elderly population [28] one could suggest *PRKN* mutations are associated with increased risk of LB pathology. Rodent models of *SNCA* [30], *LRRK2* [10], *DNAJC13* [135], *PRKN* [70], and *PINK1* [19] are associated with α-synuclein aggregation. In contrast, there have been no studies investigating α-synuclein in rodent models of *PARK7*, *TMEM230*, or *LRP10*.

**Table 1:** The genes causing rare monogenic disorders that are associated with increased risk of developing LB pathology

Mutation	Gene function	Phenotype	Onset	Characteristic pathology	α-Synuclein pathology
Familial PD					
SNCA	Encodes the protein α-synuclein, the function of which remains elusive but is thought to have a role in membrane maintenance or regulation of synaptic vesicles [13, 76]	Typically psychiatric features with more rapid decline than idiopathic PD [66]	Variable, from 18 to 77 years old	Neuronal loss in substantia nigra, locus coeruleus and hippocampus. LB pathology, with glial pathology also observed in A30P and G51D cases, and axonal spheroids in E46K [104]	Detailed in characteristic pathology
LRRK2	Encodes the protein LRRK2, a tyrosine kinase that strongly interacts with the Rab family of proteins, which are involved in vesicle transport and tethering [117, 118]	Similar to idiopathic PD [63]	Late onset	Neuronal loss in substantia nigra, often also in locus coeruleus [103]	LBs in approximately one half of cases [98]
DNAJC13	Encodes a protein which is present on endosomal membranes and is thought to have a role in intracellular trafficking [41]	Slowly progressive parkinsonism [103]	Late onset	Limited data from four cases describes LB pathology similar to idiopathic PD in three cases, with tau pathology in the fourth [131]	Detailed in characteristic pathology
PRKN	Encodes the protein parkin that ubiquitinates mitochondria for mitophagy [107]	Similar to idiopathic PD but lower rates of cognitive impairment [11]	Early onset	Neuronal loss in substantia nigra and locus coeruleus [103]	LBs in approximately one third of cases [103]
PINK1	Encodes a protein that is involved in mitochondrial quality control alongside parkin [37]	Similar to idiopathic PD but lower rates of cognitive impairment [51]	Early onset	Limited data, but reports in three cases describe neuronal loss in substantia nigra but sparing of locus coeruleus, alongside LBs or neurites in all cases, though typically of limited distribution [100, 116, 127]	Detailed in characteristic pathology
PARK7	Encodes the protein DJ-1, the function of which is largely unknown but is thought to protect the cell from oxidative stress [96]	Similar to idiopathic PD [97]	Early onset	Limited data from one case suggests substantia nigra and locus coeruleus neuronal loss, alongside widespread and severe LB pathology with additional features, such as α-synuclein-immunoreactive axonal spheroids [126]	Detailed in characteristic pathology
TMEM230	Encodes a transmembrane protein implicated in intracellular trafficking and autophagy [65]	Similar to idiopathic PD [24]	Late onset	Neuronal loss in substantia nigra. LB pathology typical of PD [24]	Detailed in characteristic pathology
LRP10	Encodes a protein that is thought to be a lipoprotein receptor [121]	Often similar to idiopathic PD, but also reports of atypical phenotypes reminiscent of frontotemporal dementia, amyotrophic lateral sclerosis and progressive supranuclear palsy [95]	Late onset	Neuronal loss in substantia nigra, widespread and severe LB pathology in 5/6 case studied and Alzheimer-type pathology in the remaining case [95, 129]	Detailed in characteristic pathology
NBIA					
PLA2G6 (PLA2G6-associated neurodegeneration)	Encodes a protein thought to be involved in lipid membrane homeostasis and remodelling [12]	Infantile and teenage forms involve developmental delay/regression, muscle hypotonia and spasticity. Adult-onset forms are characterised by dystonia and early-onset parkinsonism [7, 45, 61]	Infancy, adolescence or adulthood	Iron deposition and neuronal loss, particularly in globus pallidus and substantia nigra, alongside widespread neuroaxonal spheroid formation and neuronal loss [68]	LB pathology throughout the brain in every reported case, including children [90]
C19orf12 (Mitochondrial membrane-associated neurodegeneration)	Encodes a protein with putative roles in mitochondrial homeostasis and lipid metabolism [5]	Dysarthria, spasticity and dystonia, in addition to neuropsychiatric symptoms of dementia, depression and hallucinations [52]	Childhood or early adulthood	Iron deposition and neuronal loss, particularly in globus pallidus and substantia nigra, alongside widespread neuroaxonal spheroid formation and neuronal loss [68]	LBs throughout the brain in every reported case [44, 49, 54]
LSDs and lipidoses					
GBA1 (Gaucher disease)	Encodes the lysosomal enzyme glucocerebrosidase, that degrades lipids such as glucosylceramide [26]	Infantile and childhood forms characterised by intellectual disability, muscle hypotonia and failure to thrive. Later-onset forms involve psychiatric disturbance, cognitive dysfunction and parkinsonism [111]	Infancy or adulthood	Enlarged macrophages containing glucocerebrosidase substrates termed Gaucher cells throughout the body, particularly in spleen, liver and lung, and widespread astrogliosis and neuronal loss in central nervous system [111, 134]	LB pathology in all cases with parkinsonism phenotype [84, 134]
HEXA/HEXB (GM2 gangliosidoses)	Encode subunits of the lysosomal enzyme hexosaminidase that degrades GM2 gangliosides [15]	Tay–Sachs disease (HEXA) and Sandhoff disease (HEXB) characterised by seizures, developmental regression, hypotonia and dementia [31, 114]	Infancy or early childhood	Vacuolation of neurons and glia, lipid storage [124]	Limited data but one study of two Sandhoff disease and one Tay-Sachs disease demonstrated spherical α-synuclein accumulations throughout the brain of all cases [124]
NAGLU (Sanfillipo Type B)	Encodes the lysosomal enzyme α-*N*-acetylglucosaminidase that degrades heparan sulphate [3]	Language delay, behavioural changes such as aggression, and development delay [138]	Childhood	Neuronal swelling in cortex, dendritic swelling, dilated white matter perivascular spaces in white matter, thalamic neuronal loss, and loss of pigmented substantia nigra neurons [47]	Limited data from three cases demonstrated spherical α-synuclein aggregates in brainstem and, more variably, cortical areas [47]
NPC1 (Niemann-Pick Type C1)	Encodes the lysosomal membrane protein NPC1 [136]	Hepatosplenomegaly, developmental delay, ataxia, hypotonia, and saccadic abnormalities [123]	Childhood and adolescence	Widespread accumulation of gangliosides and unesterified cholesterol in neurons, meganeurite formation [139]	Limited data from 13 cases suggests pre-LB α-synuclein aggregates in 9 cases and normal LBs in 3 cases [17, 99]
CTSD (Neuronal ceroid lipofuscinosis type 10)	Encodes the lysosomal protease cathepsin D [79]	Decelerated head growth during last trimester, low-set or under-developed ears, intractable seizures and apnoea at birth leading to death within days of birth [112]	Antenatal	Severe neuronal loss and gliosis in cortex and cerebellum, accumulated storage materials in glia [112]	Limited data from three infant cases demonstrated spherical α-synuclein deposits reminiscent of LBs in thalamus, cortex and cerebellum [21]
GALC (Krabbe disease)	Encodes the lysosomal enzyme β-galactocerebrosidase that degrades galactosylceramide [43]	Hyperirritability, hypersensitivity to external stimuli, hypotonicity and psychomotor regression [22, 46, 71]	Mostly infancy, some later-onset cases have been reported	Spongiosis of white matter, swollen lipid-laden and multi-nucleated globoid cells [57]	Limited data from three infantile cases reported spherical Thioflavin-S- and α-synuclein- positive inclusions reminiscent of LBs in cortical regions [113]
GLA (Fabry disease)	Encodes the lysosomal enzyme α-galactosidase that degrades glycolipids and glycoproteins [86]	Excruciating pain in extremities and angiokeratoma, leading to cardiac and renal complications [39, 53]	Variable, from early childhood to adulthood	Limited studies report lipid-laden deposits in neurons and swollen macrophages throughout the brain [69, 125]	Limited data from two cases evaluated for α-synuclein reported one case with widespread LB pathology and the other unaffected [23]
Others					
POLG (mitochondrial disease)	Encodes mitochondrial polymerase gamma, a nuclear-encoded DNA polymerase for mitochondrial DNA [48]	Variable syndromic presentations, most of which include epilepsy, ataxia and myopathy [120]	Variable, from infancy to adulthood	Variable, but often necrosis of occipital regions or laminar cortical necrosis, alongside loss of cerebellar Purkinje cells [28]	Limited data from a prospective series of four cases over the age of 50 reported typical LB pathology in brainstem and limbic regions, with cortex more variably affected, in two cases [28]
RAB39B (RAB39B-associated neurodegeneration)	Encodes a protein involved in intracellular trafficking [40]	Cognitive impairment, microcephaly, autistic spectrum disorder, parkinsonism [40, 133]	Early onset	Subcortical atrophy, prominently affecting the substantia nigra and globus pallidus, mineralisation and iron deposition in the basal ganglia [36, 110, 133]	LBs have been reported in all cases in which α-synuclein has been histologically investigated [36, 133]

### NBIA

Iron is present throughout the brain, where it is involved in several important functions including energy production, DNA repair, phospholipid metabolism and myelination [20, 33]. NBIA are a range of disorders characterised by cerebral iron accumulation, giving rise to a range of neurodegenerative diseases that are distinguished into sub-types on the basis of the gene that causes them. Irrespective of underlying genetic cause, spasticity and dystonia are typical presenting features, and onset is often in early life, including infancy [7, 104]. A comprehensive review of the genetics, pathophysiology and neuropathology of NBIA has already been conducted [104]; therefore, the present discussion will focus only on NBIA disorders in which LBs have been reported: *PLKA2G6*-associated neurodegeneration (PLAN) [90] and mitochondrial membrane protein associated neurodegeneration (MPAN) [44, 49, 54] as described in Table 1. LBs are an invariant finding in every neuropathological case reported in the literature, including in a PLAN case aged 8 and an MPAN case aged 23, much younger than the earliest age at which incidental LBs have been reported in control populations, which is typically approximately 60 years old [34, 88]. A rodent model of *PLA2G6* knockout demonstrated widespread α-synuclein aggregation, particularly on mitochondrial membranes [122], though there are no studies of α-synuclein aggregation in rodents with *C19orf12* mutation or deletion to our knowledge.

### Lysosomal storage disorders and lipidoses

Lysosomal storage disorders (LSD) are caused by mutations in the genes that encode either lysosomal enzymes or membranes, resulting in impaired lysosomal breakdown of cellular components and accumulation of waste products within cells, particularly those within the central nervous system [108]. Lipidoses are disorders characterised by altered lipid metabolism, often by mutations in lysosomal enzymes, resulting in accumulated lipids in vulnerable cells [124]. LSD and lipidoses typically present in infancy or early childhood, though some cases can occur up to adulthood, and whilst clinically heterogeneous are usually characterised by developmental delay or regression, and hypotonia [31]. Detailed neuropathological reports from many of the rare mutations causing LSD and lipidoses are lacking, so the present discussion will focus on those with reported α-synuclein pathology: Gaucher disease [84], GM2 gangliosidosis [124], Sanfilippo syndrome [47], Niemann–Pick disease Type CI [99], neuronal ceroid lipofuscinosis type 10 [21], Fabry disease [23] and Krabbe disease [113], as described in Table 1.

Many lipidoses are plausibly linked to the aggregation of α-synuclein as they result from loss of function of lipid-degrading enzymes, the substrates of which have been demonstrated to induce the aggregation of α-synuclein in vitro. For example, Krabbe disease results from mutations in *GALC* encoding the enzyme galactosylceramidase, resulting in the accumulation of the cytotoxic lipid psychosine which has been demonstrated to induce to fibrillization of α-synuclein in vitro [113] through direct interactions with its C-terminal region that expose the central amyloidogenic region [1]. Sanfillipo Type B results from loss-of-function mutations in the lysosomal enzyme α-*N*-acetylglucosaminidase, leading to accumulation of its substrate heparan sulphate [3], which increases the rate of α-synuclein fibrillization in a dose-dependent manner in vitro, possibly by binding the N-terminus and inducing conformational changes permissive to fibrillation [18]. The influence of lipidosis-causing genetic mutations may be two-fold, with both reduced clearance of α-synuclein due to autophagic impairments leading to a state of increased abundance of α-synuclein within cells, combined with the accumulation of lipids known to promote α-synuclein aggregation, as has been demonstrated for GBA1 [119].

**Fig. 1:**
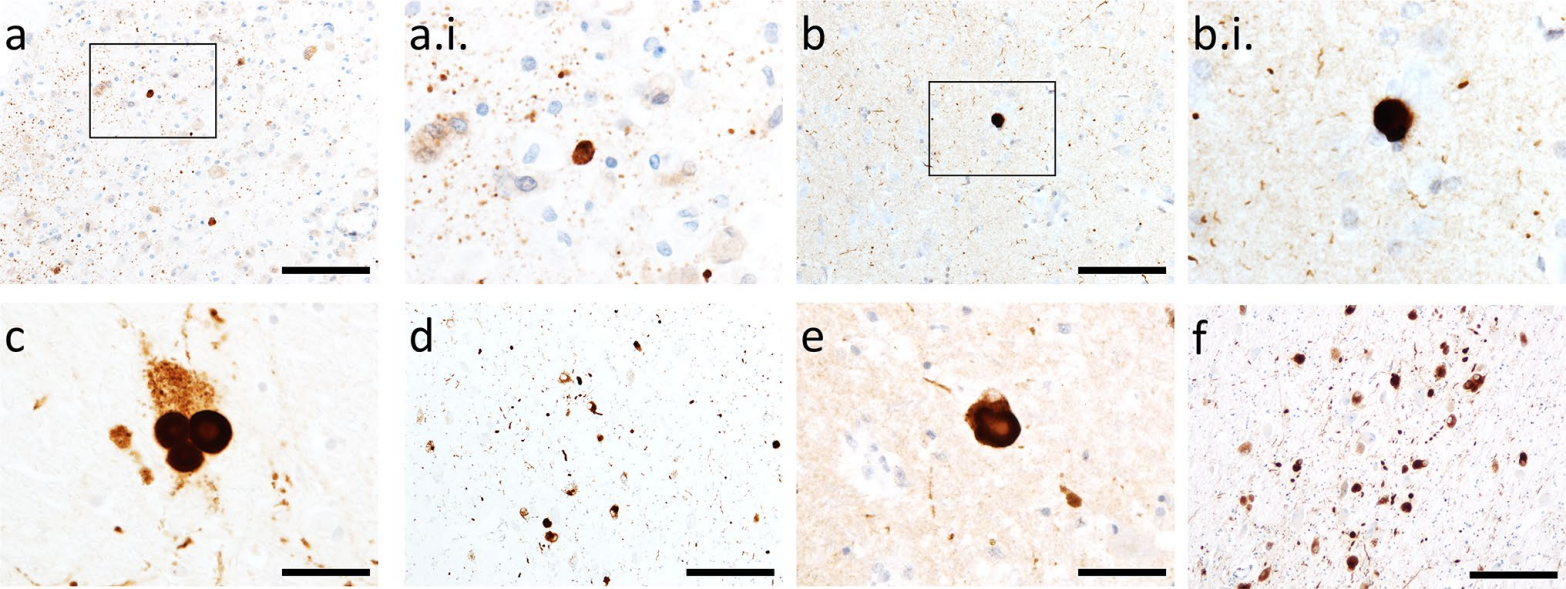
α-Synuclein immunoreactivity in rare monogenic disorders in comparison to idiopathic LB diseases. α-Synuclein-positive punctae and small, LB-like structures, in temporal cortex grey-white matter junction in a 10 month old boy with Krabbe disease (**a** and **a.i.**) in comparison to superficial pyramidal layer of temporal cortex in a 91-year-old female with dementia with LBs (**b**–**b.i.**). LBs in substantia nigra (**c**) and nucleus basalis of Meynert (**d**) of a 87-year-old female with dementia with LBs in comparison to the substantia nigra (**e**) and nucleus basalis of Meynert (**f**) of a 79-year-old male with a *POLG* mutation and longstanding progressive external opthalmoplegia taken from our previous report of LB pathology in mitochondrial disease [28]. Antibodies used were BD Transductions Clone 42 (1:1,000; **a**–**b.i.**) and Novocastra KM51 (1:250; **c**–**f**). Scale bars = 100 µm (**a** and **b**), 50 µm (**c** and **e**) and 200 µm (**d** and **f**)

It is notable that most cases in the literature have reported α-synuclein pathology in cases deceased in infancy or childhood, much earlier than incidental LBs typically develop. However, it is not clear to what extent α-synuclein pathology in LSDs and lipidoses is similar to that observed in idiopathic LBD in terms of its capacity to induce native α-synuclein to misfold, underlying the need for further studies on the ultrastructure and seed-competency of LB pathology in rare LSDs and lipidoses. Representative images of α-synuclein immunoreactivity in an infantile Krabbe disease case obtained prospectively can be found in Fig. 1 (A–B.i.). Rodent models of Gaucher disease (*GBA1*) [56], Sandhoff disease (*HEXA*) [62], Tay–Sachs disease (*HEXB*) [14], neuronal ceroid lipofuscinosis type 10 (*CATD*) [21], Krabbe disease (*GALC*) [113], and Fabry disease (*GLA*) [83] manifest accumulated insoluble α-synuclein. In contrast, no study has yet investigated whether α-synuclein is accumulated in Sanfillipo syndrome (*NAGLU*) or Niemann–Pick Type C1 (*NPC1*) rodent models.

### Mitochondrial diseases

Mitochondrial diseases result from mutations in either nuclear or mitochondrial DNA, inducing perturbed cellular respiration and degeneration of cellular populations with the highest energy requirement [42]. Mitochondrial diseases are heterogeneous entities, even across cases with the same mutation, and age of onset, clinical presentation and neuropathological features can vary [42]. *POLG* encodes polymerase gamma, a nuclear-encoded DNA polymerase for mitochondrial DNA, mutations in which give rise to several clinical syndromes, including: Alpers–Huttenlocher syndrome (AHS), myocerebrohepatopathy spectrum (MCHS), myoclonic epilepsy myopathy sensory ataxia (MEMSA), ataxia neuropathy spectrum (ANS) and progressive external ophthalmoplegia (PEO) [120]. We have reported a higher prevalence of LB pathology in a prospective series of older mitochondrial disease cases, particularly those with *POLG* mutations, compared to a control population [28], as described in Table 1. Representative images of cortical and midbrain LBs in a 79-year-old individual with a *POLG* mutation, in comparison to an individual with dementia with LBs, can be found in Fig. 1c–f. To the best of our knowledge, no study has yet evaluated α-synuclein aggregation in *POLG* mice.

### RAB39B-associated neurodegeneration

A number of RAB39B mutations resulting in the loss of expression/function of the protein are associated with X-linked mental retardation, autistic spectrum disorder and early onset PD, as described in Table 1 [40, 133]. Although somewhat heterogeneous in terms of symptom presentation, lifelong non-progressive cognitive impairment with underlying macrocephaly is common, as is early onset PD, occurring between 10 and 50 years of age [40, 133]. In those limited cases where α-synuclein immunoreactivity has been investigated, both subcortical and cortical LBs were reported [36, 133]. Furthermore, we have recently reported RAB39B as reduced in post-mortem LBD brain tissue and sequestered into some LBs, potentially indicating a role for RAB39B in idiopathic LBD [67]. To the best of our knowledge, α-synuclein aggregation has not yet been investigated in *RAB39B* rodent models.

## Gene ontology analysis of risk genes for LB pathology

To better understand commonalities across the range of genetic disorders in which LBs are observed, we used both the Gene Ontology Resource [6, 128] with PANTHER gene enrichment software [77], and ShinyGO [38], to identify common biological processes enriched in these genes associated with LB pathology.

Analysis using PANTHER demonstrated enrichment of biological processes related to mitochondrial function (negative regulation of hydrogen peroxide-induced neuron intrinsic apoptotic signalling pathway, regulation of peroxidase activity, mitochondrion to lysosome transport, positive regulation of mitochondrial electron transport, positive regulation of mitophagy in response to mitochondrial depolarization), lysosomal degradation (regulation of peroxidase activity, mitochondrion to lysosome transport, regulation of retrograde transport endosome to Golgi, positive regulation of mitophagy in response to mitochondrial depolarization) and lipid catabolism (ganglioside catabolic process, glycosylceramide catabolic process; Table 2).

**Table 2:** Analysis with PANTHER [77] demonstrated enrichment for genes implicated in mitochondrial function

GO biological process	Fold enrichment	*P* value	FDR
Negative regulation of hydrogen peroxide-induced neuron intrinsic apoptotic signalling pathway	> 100	5.23E-06	1.11E-03
Regulation of peroxidase activity	> 100	8.71E-06	1.65E-03
Mitochondrion to lysosome transport	> 100	8.71E-06	1.61E-03
Positive regulation of mitochondrial electron transport, NADH to ubiquinone	> 100	8.71E-06	1.56E-03
Regulation of synaptic vesicle transport	> 100	8.99E-08	7.15E-05
Regulation of retrograde transport, endosome to Golgi	> 100	1.83E-05	2.69E-03
Positive regulation of histone deacetylase activity	> 100	2.43E-05	3.25E-03
Ganglioside catabolic process	> 100	3.13E-05	3.95E-03
Glycosylceramide catabolic process	> 100	3.13E-05	3.92E-03
Positive regulation of mitophagy in response to mitochondrial depolarization	> 100	4.77E-05	5.15E-03

Top ten enriched processes when ranked based on FDR are shown

**Fig. 2:**
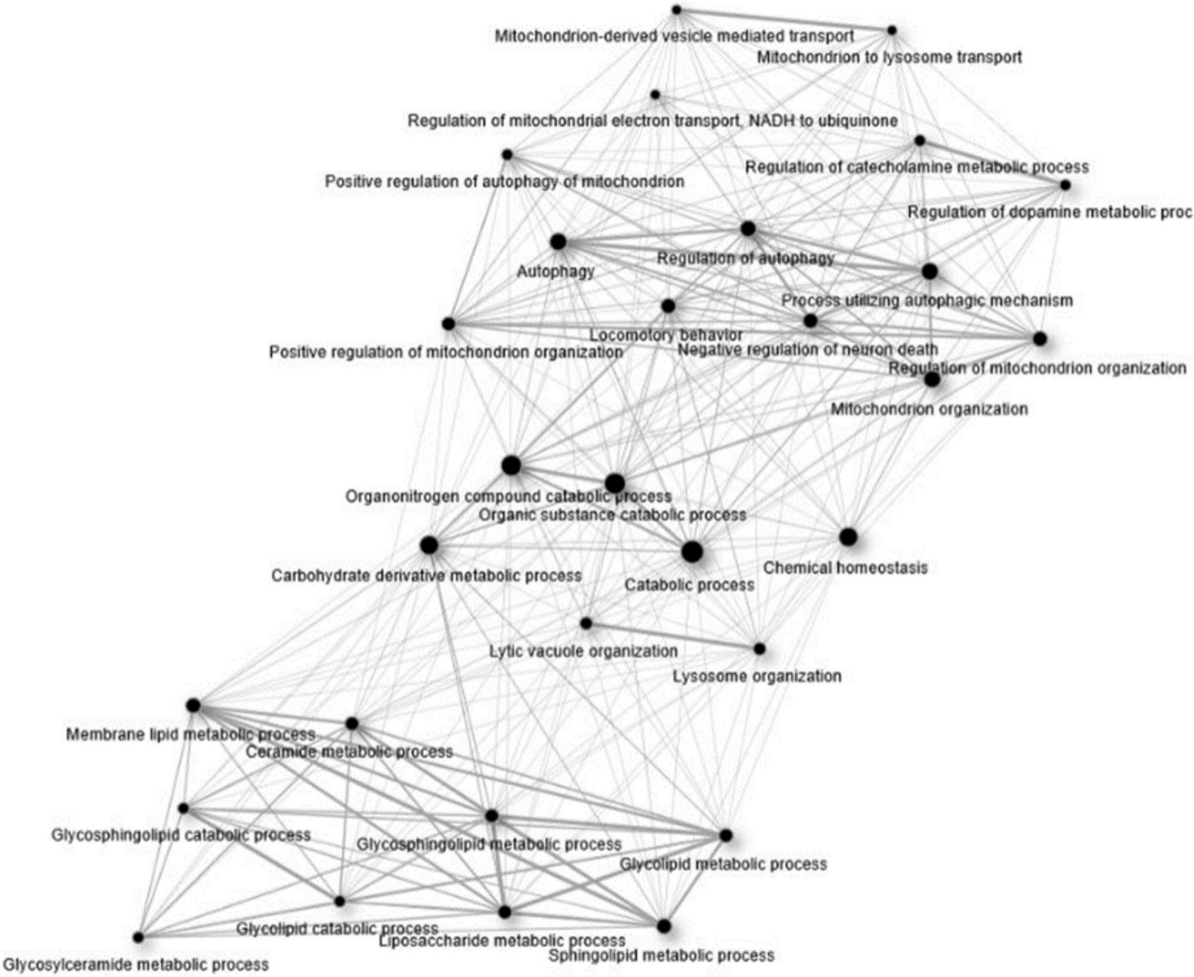
ShinyGO [38] analysis demonstrated three broad clusters into which enriched biological processes clustered: lipid metabolism, catabolic processes, and mitochondrial homeostasis and autophagy

Evaluation of the genes associated with LB pathology using ShinyGo largely confirmed the findings from PANTHER, with enriched biological processes functionally clustered around two sub-groups of mitochondrial function/autophagy, and lipid metabolism, linked by catabolism (Fig. 2).

## Lipids, lysosomes, mitochondria and LB pathology

One of the primary reasons for the ascendency of α-synuclein in LB disease is that it is responsible for mutations causing the first identified form of familial PD and is a component of LBs [88]; however, a recent study has reported that the core of LBs may be composed of lipids and surrounded by dystrophic mitochondria [109]. The novel report of LBs as having a lipid core has led to considerable debate in the field as to whether LBDs are indeed proteopathies or whether they should be considered a lipidopathy [29]. By evaluating the spectrum of monogenic disorders in which LBs are frequently observed we have continued this debate by identifying that alterations to lipid metabolism, autophagy and mitochondrial function are common pathways affected in the genetic mutations associated with LB pathology. Thus, a key question is how alterations to these distinct processes contribute to α-synuclein aggregation and the formation of LBs.

**Fig. 3:**
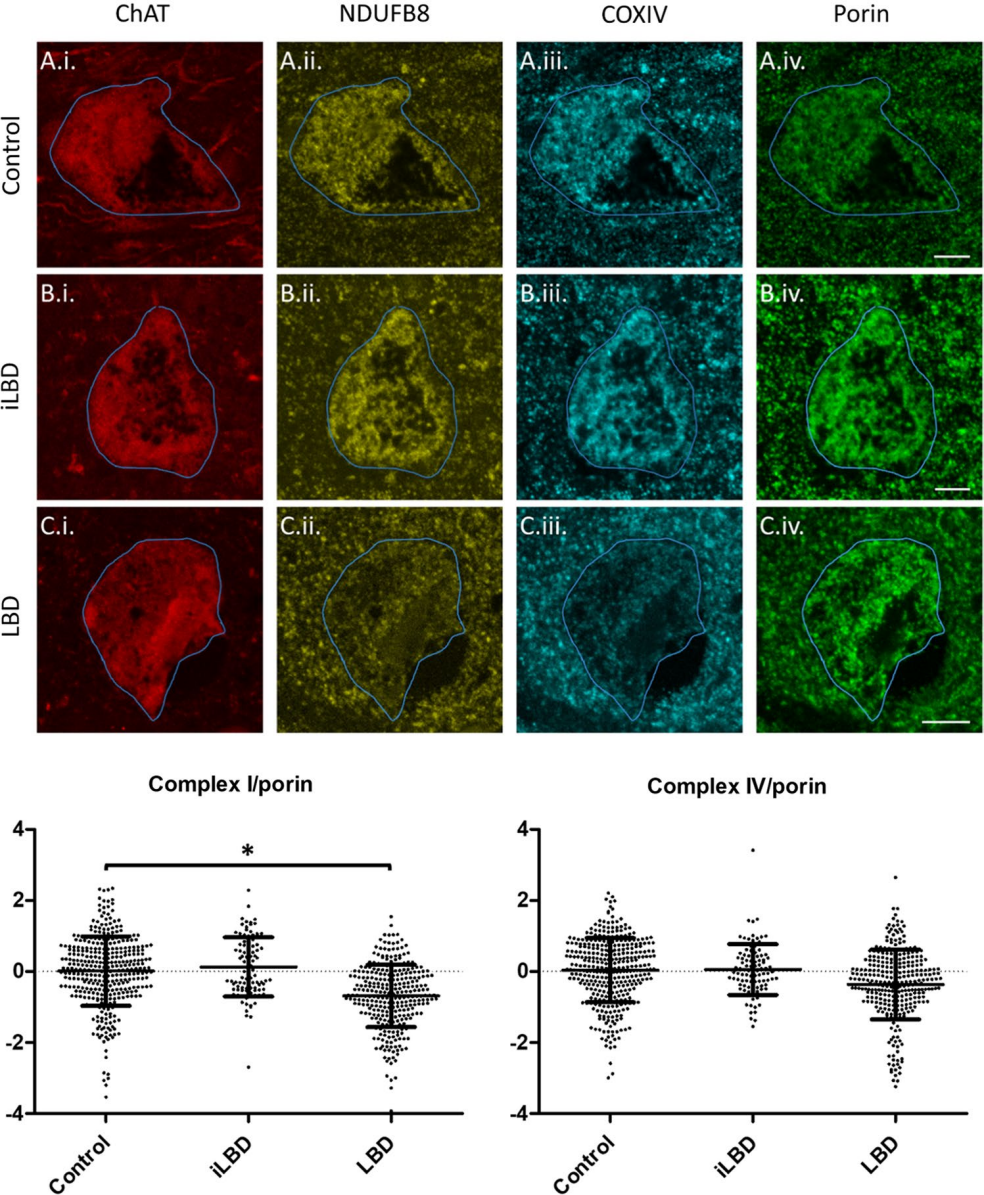
LB dementia is associated with changes to the mitochondrial respiratory chain. Representative images from our previous study in the nucleus basalis of Meynert [50] demonstrating respiratory chain subunit expression in control (**A.i.**–**A.iv.**), incidental LB disease (iLBD) (**B.i.**–**B.iv**.) and LB dementia (LBD) (**C.i.**–**C.iv.**) cases, highlighting reductions in Complex I in LBD compared to iLBD and control. As detailed in [50], sections were stained with ChAT (Sigma HPA048547, 1:100), NDUFB8 (Abcam ab110242, 1:100), COX4 (Abcam ab110261, 1:100) and VDAC1/porin (Abcam ab14734, 1:200). Scale bars = 10 µm. Dot plots show group level z scores of Complex I NDUFB8 and IV/COXIV integrated densities normalised to porin integrated density, as explained in detail in [50], from approximately 50 neurons per case (control *N* = 8, LBD *N* = 8, iLBD *N* = 2). Bars are means and standard deviation. *p < 0.05. Originally published in [50] by BioMed Central and provided here under a Creative Commons Attribution Licence 4.0

Deficits in mitochondrial energy production have long been implicated in LB diseases, from the early identification of complex I inhibitors causing parkinsonism and α-synuclein aggregation, to the identification of mutations in mitochondrial proteins causing familial PD [16]. Alterations to the mitochondrial respiratory chain are a consistent finding in LB diseases, and we have previously reported reductions in Complex I of the mitochondrial respiratory chain in cholinergic nucleus basalis of Meynert neurons in LB dementia (Fig. 3) [50]. Energy production is an attractive hypothesis to explain the potential contribution of lipid metabolism, autophagy and mitochondrial function to LB formation, as catabolic processes were implicated in the GO analysis. Cellular energy in the form of adenosine triphosphate (ATP) is primarily produced in mitochondria, where glucose is broken down by glycolysis to form pyruvate, which is then converted to the metabolic intermediate acetyl-Coenzyme A (acetyl-CoA) to enter the citric acid cycle and mitochondrial respiratory chain to generate ATP [87]. Deficient energy production may contribute to α-synuclein aggregation through excessive production of reactive oxygen species (ROS), leading to the accumulation of oxidised α-synuclein that is more resistant to degradation [74]. It has also been suggested that increasingly oxidised intracellular environments may lead to reductions in binding partners of α-synuclein, increasing levels of unbound α-synuclein, culminating in its aggregation [102].

Autophagy is a critical process that degrades mitochondria, lipids, and many other organelles or macromolecules, the accumulation of which can significantly impair cellular functioning, and there are multiple lines of evidence from model systems and post-mortem tissue indicating it is deficient in LB disease [55]. Whilst autophagic deficits have intuitive appeal for promoting α-synuclein aggregation by impeding its degradation, it is not clear how deficits in a relatively non-specific process like autophagy would selectively induce α-synuclein accumulation and not that of other aggregation-prone proteins. A recent study reported that LBs contain numerous lipids, dystrophic mitochondria and other organelles, and thus one could speculate they are accumulating and compartmentalising damaged organelles and macromolecules as a protective mechanism in the context of deficient autophagic processes [109]. In this context, α-synuclein aggregation could be a stereotyped response to autophagy failure with the aim of protecting the cell from the deleterious effects of accumulated macromolecules and organelles such as mitochondria. Consistent with a putative protective role for LBs, we have previously reported that neurons of the nucleus basalis of Meynert harbour mitochondrial respiratory chain deficits and increased levels of mitochondria in LB dementia, but that neurons with LBs have fewer deficits [50]. However, autophagic processes play a critical role in mitochondrial quality control through selective mitochondrial degradation termed mitophagy, and there is evidence to suggest this is impaired in LB diseases, thus suggesting autophagic deficits could induce accumulation of dysfunctional mitochondria [32, 91]. Furthermore, our recent work indicates NAD(H), an essential cofactor for mitochondrial metabolism, is depleted by selective inhibition of autophagy, demonstrating that deficient autophagy induces mitochondrial dysfunction [105]. In summary, whilst autophagy may relate to α-synuclein aggregation by impeding its degradation, this seems a sub-optimal explanation given the likely impact this would have on aggregation-prone proteins beyond α-synuclein. Therefore, it seems more likely that the impact of autophagy impairments on α-synuclein aggregation may implicate other cellular processes, such as a hypothesised protective role or an impact upon mitochondrial function or quality control, and that this underlies the apparent selective accumulation of α-synuclein in the context of autophagic deficits.

Lipid homeostasis is vital for cellular health as the accumulation of lipids within cells induces the cellular stress response and lysosomes play a key role in preventing lipid accumulation by degrading lipids in a selective autophagic process termed lipophagy, and also by acting as a nutrient sensor to regulate lipophagy [58]. Thus, autophagy is critical for the maintenance of lipid homeostasis. However, mitochondria are also a major site of intracellular lipid degradation as they are the site of fatty acid β-oxidation, the catabolic process that breaks down fatty acids to generate acetyl-CoA [80]. Intracellular accumulation of lipids leads to excessive production of ROS and decreased mitochondrial biogenesis, inducing a state of decreased mitochondrial respiratory function and diminished ATP production, in turn leading to decreased mitochondrial degradation of lipids and further lipid accumulation [59]. Accumulation of lipids, or at least dyshomeostasis amongst lipid species in the brain, may have a direct influence on α-synuclein aggregation as lipid membrane surfaces have been proposed as a potential site of α-synuclein aggregation, with the degree of membrane binding inversely proportional to the propensity of α-synuclein to polymerise [130]. Furthermore, exposure of α-synuclein to polyunsaturated fatty acids such as arachidonic acid and linoleic acid has been demonstrated to induce rapid aggregation of α-synuclein [35, 93], and we have previously demonstrated that α-synuclein aggregation also promotes accumulation of lipids [89], potentially creating a cycle of increasing lipid and α-synuclein aggregation. Therefore, it is plausible to suggest that accumulations of particular lipids contribute to the aggregation of α-synuclein, perhaps by inducing conformational changes that are permissive to aggregation [81]. It is notable that accumulation of lipids within cells could occur secondarily to diminished mitochondrial capacity to perform β-oxidation or impaired autophagic degradation of lipids.

**Fig. 4:**
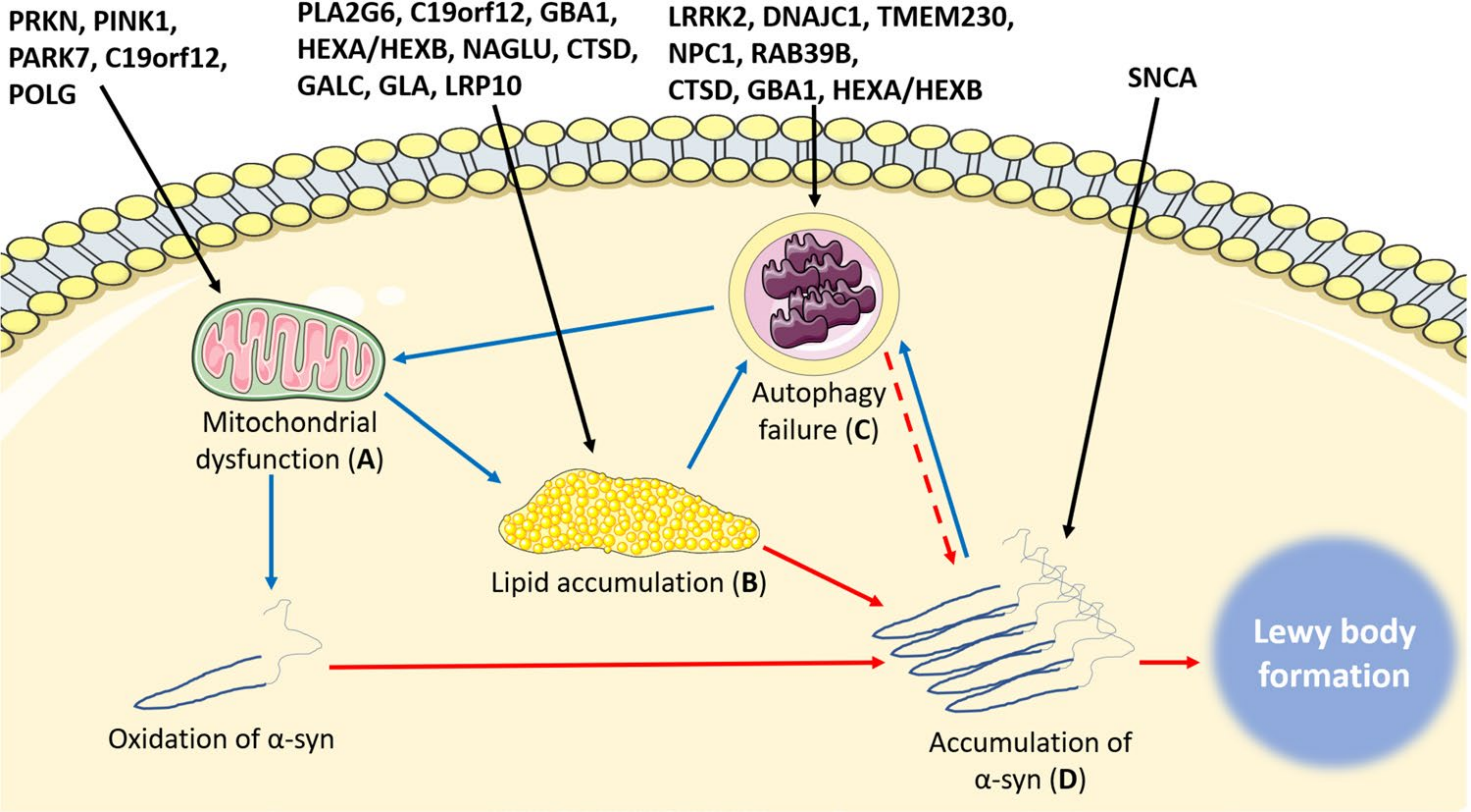
Multiple pathways leading to Lewy body formation. **a** Mutations causing mitochondrial dysfunction may contribute to Lewy body formation by increasing oxidation of α-synuclein, leading to aggregation and eventual formation of a Lewy body. **b** Mutations in genes encoding lipid-degrading enzymes such as GALC, GBA and CATD can directly lead to increased levels of lipid substrates known to be permissive to α-synuclein aggregation, including psychosine, glucosylsphingosine and heparan sulphate, respectively. Alternatively, mitochondrial dysfunction (**a**) leads to increased abundance of lipid droplets known to facilitate α-synuclein aggregation. Elevated levels of lipids are likely to overwhelm autophagic mechanisms within neurons, leading to autophagy failure after sustained elevations in lipid species (**c**). **c** Autophagy failure induces mitochondrial dysfunction (**a**) by impeding mitochondrial quality control by reductions in mitophagy, and potentially also leads to α-synuclein aggregation by reduced turnover (dashes). (**d**) Accumulation of α-synuclein may occur directly due to disassembley of tetramers into aggregation-prone monomers or increased abundance of α-synuclein protein, or indirectly through (**a**), (**b**), or (**c**). Increased accumulation of α-synuclein over time leads to assembly into Lewy bodies. Black lines indicate the mechanism directly affected by specific mutations, blue lines indicate indirect influences on α-synuclein aggregation through interactions between mechanisms, and red lines indicate direct influences on α-synuclein aggregation

Given the interrelated nature of lipid homeostasis, mitochondrial function and autophagy, it would be possible to make a case for any one of these three aspects underlying α-synuclein aggregation in LBD, either directly or indirectly (Fig. 4). However, such reductionism overlooks the dynamic nature of cellular metabolism, and thus one could speculate that these impairments may act in concert to drive vulnerability to LB formation. Such a triad of impairments could potentially explain why cell death precedes LB formation in the substantia nigra [78], and neuronal loss occurs in regions without LB pathology in PD [4], as factors that govern cellular vulnerability to LB formation and cell death would likely differ between distinct neuronal sub-types. For example, if LB formation was driven by lipid dyshomeostasis, but cell death by mitochondrial dysfunction, then LBs would form first in neurons that normally have highest levels of the fatty acids permissive to α-synuclein aggregation and cell death would occur first in neurons with the highest energy demands. It is plausible to suggest that the neuronal sub-class with the highest energy demands may not be that with the highest levels of fatty acids permissive to α-synuclein aggregation. Such a hypothesis does not preclude LBs being deleterious for cellular health, but rather would suggest they occur as a result of, and alongside, other changes that are likely to impact neuronal health.

## Conclusion

The present review has discussed a range of rare monogenic diseases that are disproportionately affected by LB pathology, on the basis that commonalities in biological pathways in which the protein products of the affected genes participate may provide insights into LB formation in idiopathic LB disease. In summarising the current published literature on α-synuclein pathology in a diverse array of monogenic disorders, followed by GO analysis of the genes implicated in these disorders, autophagy, lipid metabolism and mitochondrial function emerge as common biological pathways. Impairments to these pathways could occur as a trio of impairments that underlie both the selective nature of LB formation in distinct neuronal populations and the selectivity of neurodegeneration in LB disease. It is important to note that there are many metabolic disorders, the majority of which do not seem to be vulnerable to LB formation, and thus it seems likely that there are specific pathways involved that are permissive to α-synuclein aggregation, rather than general alterations to cellular metabolism.

Future studies are warranted to better characterise the structure of LBs, to further elucidate the lipid components within LBs and the lipid species that are most abundant. Such studies may highlight aspects of lipid metabolic pathways that may be altered and give rise to LB pathology, directing future studies to explore mechanistic links between such dyshomeostasis and α-synuclein aggregation. Furthermore, better characterisation of cellular metabolism, and how it is affected in LBD post-mortem brain tissue is also warranted, to better model how it may contribute to LB formation. For example, there is considerable evidence of mitochondrial respiratory chain dysfunction in LBD, but relatively little is known about peroxisomal function, despite their role in degrading very long chain fatty acids for mitochondrial β-oxidation and scavenging ROS placing them at a potentially pivotal juncture between lipid catabolism and mitochondrial energy production [132]. The role of processes other than α-synuclein aggregation in LBDs is critically important for the field as considerable expense and effort is being expended targeting α-synuclein aggregation as a potential disease-modifying therapy for LBD. However, if LB formation is itself a by-product of other altered cellular processes such as metabolic balance, then it would seem more likely to be effective to target amelioration of cellular metabolism than α-synuclein aggregation.

A limitation of the present review is that some of the disorders covered are exceptionally rare, with neuropathological data limited to a small number of reports. Therefore, whilst the present review summarises the current state of knowledge on rare monogenic disorders disproportionately vulnerable to LBs, it is likely that there are further similar conditions vulnerable to LB pathology that have not been subject to detailed neuropathological evaluation of α-synuclein. Furthermore, there may be a reporting bias in the prevalence of LBs in some conditions, as the presence of an age-associated feature like LBs in young individuals may be more likely to be reported than more banal findings. It is also not clear whether α-synuclein accumulations in many of the rare monogenic disorders has similar attributes to that observed in idiopathic LB disease, such as the propensity to seed aggregation of native α-synuclein to spread in a ‘prion-like’ manner, or its contribution to clinical phenotype. The present review underlines the importance of further study of α-synuclein aggregation in rare diseases to better understand the aetiology of LB formation in idiopathic LB disease and its biological relevance to the pathobiology of the rare diseases in which it is also observed.
